# Algorithmic management, preferences for autonomy/security and gig-workers' wellbeing: A matter of fit?

**DOI:** 10.3389/fpsyg.2023.1088183

**Published:** 2023-02-24

**Authors:** Bruno Felix, Diego Dourado, Valcemiro Nossa

**Affiliations:** Department of Accounting and Business Administration, Fucape Business School, Vitória, Brazil

**Keywords:** algorithmic management, gig-work, P-O fit, paradox mindset, gig economy

## Abstract

The objective of this study was to understand how gig-workers interpret the effects of their laboral activity on their wellbeing. We developed a grounded theory based on interviews with 57 Brazilian gig-workers. The results show that (1) workers and gig-work organizations have preferences for work relationships with more autonomy or security; (2) when there is a congruence of preferences, the worker experiences greater wellbeing, and when the preferences diverge, there are episodes of preference violations that, when repeated, reduce worker wellbeing; and (3) however, not everything is a matter of fit: when both individuals and organizations have the same preference (for example, for more autonomy and less security), worker wellbeing may be vulnerable to abuse, for example, in terms of an unsustainable workload. Our study draws attention to an integrated discussion of the benefits and harms of algorithmic management, which allows overcoming a polarized view in which it would be seen only as beneficial or harmful to workers.

## 1. Introduction

A new type of work termed gig-work has recently emerged in our constantly changing labor market. Gig-work comprises service provider platforms that connect consumers and on-demand workers through the use of algorithms that control and mediate workflow (Duggan et al., 2020). In this work model, employment relations are conducted by algorithmic management, which is a management system performed by self-learning algorithms that make decisions regarding the relationship with workers (ex.: Uber) (Stewart and Stanford, 2017). This form of employment relation is criticized for making employment relations precarious due to the lack of guarantees and security for workers (Harvey et al., 2017). Nevertheless, the number of workers who have adhered to this new way of working is increasing, and several report high levels of satisfaction (Berger et al., 2019). Thus, many workers have been asking themselves about the beneficial and harmful effects of algorithmic management for their wellbeing (e.g., Stewart and Stanford, 2017; Chen et al., 2019).

Previous studies have shown mixed results regarding the effects of algorithmic management on worker wellbeing (Malin and Chandler, 2017; Duggan et al., 2020). While some criticize the precariousness of organizational security of workers' rights, others praise the positive potential of autonomy that this management model generates for individuals (Hall and Krueger, 2018; Berger et al., 2019; Petriglieri et al., 2019). For example, Petriglieri et al. (2019), Stewart and Stanford (2017) and Cram et al. (2022) state that, in the absence of a relationship that offers greater security conditions, workers experience high emotional stress, anxiety and lower job satisfaction. In contrast, some authors claim that this form of work allows workers to experience greater flexibility regarding schedules, intensity and type of work they want to perform (Chen et al., 2019).

Despite the growing literature on algorithmic management and its effects on the wellbeing of employees (Duggan et al., 2020), the approaches presented are far from integrated. Thus, adopting an interpretive perspective (Stryker, 1980), in which reality is socially constructed, it is suggested here that both interpretations—that there are harms and benefits to worker wellbeing—coexist and need to be simultaneously considered. However, there is a lack of studies seeking to reconcile both approaches in their explanations for why gig-workers consider this modality beneficial or detrimental to their wellbeing. To fill this gap, the objective of this study was to understand how gig-workers interpret the effects of this activity on their wellbeing.

Here, we argue that, for workers laboring under algorithmic management, worker wellbeing is explained by a fit between individual and organizational preferences related to the degree of worker autonomy and security. However, when both individuals and organizations have the same preference (for example, for more autonomy and less security), worker wellbeing may be vulnerable to abuse, for example, in terms of an unsustainable workload.

This study has contributions for the understanding of the impacts of algorithmic management on worker wellbeing. We integrate the discussion of the benefits and harms of algorithmic management, which allows overcoming a polarized approach in which it would be seen only as beneficial or harmful to workers (Duggan et al., 2020). Also, by adopting a fit perspective for the gig-worker wellbeing phenomenon (Cable and Judge, 1996), we draw attention to the fact that the construction of a sense of wellbeing may be a result not only of the content of the relationship itself but specially from a congruence of interests between those involved. Finally, we contribute to the literature on paradoxes by discussing not organizational (Lewis, 2000; Felix, 2020) or individual (Miron-Spektor et al., 2018; Cavalcanti et al., 2022) paradoxical characteristics but rather how individual paradoxical goals can be achieved through a paradoxical relationship between individuals and organizations.

## 2. Literature review

### 2.1. Algorithmic management in gig-work

The use of technology has become increasingly present in contemporary society, and one of its specific resources has transformed the way we consume and work: the implementation of algorithms to mediate the relationship between social actors (Wood et al., 2019). An algorithm is a computational formula that allows autonomous decision-making based on statistical models or decision rules, without an individual interfering in the decision-making process (Göttel, 2021; Parent-Rocheleau and Parker, 2022). It is characterized as sequential instructions that guide the decisions of a computational system within a set of rules and steps to achieve a given task (Duggan et al., 2020; Benlian et al., 2022).

Because algorithms are operated without the visible interference of humans and are based on objective and mathematical criteria, they tend to be seen as correct and reliable (Lee et al., 2015; Angrave et al., 2016). In the context of gig-work, which are service provider platforms that use algorithms to control and mediate the work of on-demand workers, algorithms connect consumers and service providers (Duggan et al., 2020). They are also used to perform tasks traditionally performed by human resources professionals, such as assigning activities and evaluating worker performance (Rosenblat, 2018). This use has been conceptualized as algorithmic management, a control system in which self-learning algorithms are given the responsibility for making and implementing decisions associated with employment relations (Duggan et al., 2020).

Algorithmic management has five central characteristics: constant tracking of workers' behavior, constant performance evaluation, automatic implementation of decisions, workers' interaction with a “system” rather than humans, and low transparency (Möhlmann and Zalmanson, 2017). While traditional systems are founded on long-term relationships based on trust, in algorithmic management this becomes impossible, since, given the high volume of data processed, there is a constant mapping related to the behavior of workers (Göttel, 2021). In addition to analyzing data on workers' behavior, algorithmic management also maps their performance. Its results are evaluated in a comparative way, so that performance anomalies are treated automatically or, in some cases, by humans (Duggan et al., 2020). In general, algorithms calculate decisions and implement them by themselves, which leads to the understanding that “algorithms do things” (Rosenblat, 2018). This process leads to a recurring perception of lack of support to clarify or question issues related to people management (Berger et al., 2019). Despite having all the elements to present workers with high transparency regarding the processes that led to certain decision-making regarding people management, transparency in algorithmic management tends to be lower than that practiced in traditional models of management (Parent-Rocheleau and Parker, 2022). This occurs due to the high complexity of the calculations performed to support the decisions, and to a reduced interest of organizations in disclosing information regarding the decision-making processes performed by the algorithms (Meijerink and Bondarouk, 2023).

Given this emerging context, what are the consequences of implementing algorithmic management to gig-worker wellbeing? If there is a relatively homogeneous view in terms of the benefits of this model for entrepreneurs (Duggan et al., 2020), the same is not true from the perspective of workers (Gandini, 2018; Meijerink and Bondarouk, 2023). On the one hand, there are those who defend the view that algorithmic management leads to the precarization of employment relations, resulting from a reduction in their security (Malin and Chandler, 2017; Griesbach et al., 2019). On the other hand, there is also the idea that this model provides more autonomy to workers, who can define their work schedules and rhythm according to their needs (Berger et al., 2019). Next, we explore both perspectives in greater detail.

### 2.2. Worker wellbeing in gig-work: The role of security

In the literature on Organizational Behavior and Human Resource Management, there is a dominant view that it is the role of employing organizations to promote an adequate level of worker physical and psychological security (Lee, 2018; Toth-Kiraly et al., 2021). However, according to these perspectives, gig-workers experience work relationships in which their security is precarious and, as a result, such relations are deleterious to worker wellbeing (Duggan et al., 2020; Chan, 2022). By promoting worker invisibility and discouraging worker organization around collective forces, workers lose their voice, and a favorable space is opened for an unbalanced power relationship (Webster, 2016).

The structures of algorithmic management and gig-work enable employers to access relatively inexpensive labor without great academic or professional qualifications (Wright et al., 2017) and to develop asymmetric information and power relationships that lead to the control of workers (Rosenblat and Stark, 2016). Such relationships distance themselves from more traditional labor practices by establishing a social exchange in which long working hours (Stewart and Stanford, 2017), higher levels of uncertainty in relation to the future (Wood et al., 2019) and reduced support for worker's belonging- and self-esteem-related issues (Petriglieri et al., 2019) are often accompanied by a lack of remuneration compatible with exposure to such risk (Malin and Chandler, 2017).

### 2.3. Worker wellbeing in gig-work: The role of autonomy

Despite the dominance of studies in the Human Resources Management literature highlighting the role of security, this is not a homogeneous current in the impacts of gig-work on worker wellbeing. Some studies claim that individuals can achieve increased levels of wellbeing through the greater autonomy provided in the gig-work modality (e.g., Berger et al., 2019; Kost et al., 2020; Möhlmann et al., 2021). The logic of this argument is as follows: the security offered to workers in more traditional employment relations has a price. To obtain labor rights that offer greater stability, predictability and security against accidents and other health risks, individuals experience significant losses in terms of flexibility in their work pace and pay (Wheatley, 2017). Thus, gig-workers would be attracted by the offered level of workflow flexibility, financial compensation and the fact that hourly earnings do not vary much with the number of hours worked (Hall and Krueger, 2018).

Studies in this line of argument show that Uber drivers, for example, appreciate flexibility in the pace of work, even working for longer periods than cab drivers (Hall and Krueger, 2018; Chen et al., 2019; Unruh et al., 2022). In addition, they also see the possibility of reasonably controllable fluctuation in their income as desirable, since they can intensify their work activity in the months of greatest need (Hall and Krueger, 2018). In these studies, it is also argued that the reduction or elimination of barriers to entry and the promotion of lower taxation for gig-work platforms democratized access to work (Berger et al., 2018; Ravenelle, 2019).

In this study, the arguments that the gig-work model brings harms (via violations of security needs) and benefits (by meeting autonomy needs) are integrated. Next, two theoretical lenses that act as sensitizers in the development of the present study are presented: P-O fit and paradox theory.

### 2.4. Person-organization fit

The literature on gig-work suggests that an essential condition for individuals to experience wellbeing in this type of work is a congruence between their needs and preferences and the values promoted in a given form of work (Berger et al., 2019). Individuals have different levels of need for security and autonomy (Felix and Cavazotte, 2019), and organizations also have values that guide them toward proposing work relationships based on different degrees of predictability/stability or risk-taking/risk-sharing (Gehman et al., 2019). Thus, it is possible that, in a specific relationship, both the individual and the employing organization have a preference for employment relations more grounded in the value of security or autonomy. However, it is also possible that they have divergent preferences about the degree to which relationships should be more secure or free. Thus, employment relations between individuals and organizations may present (in)congruence regarding their preferences as to the degree to which employment relations should protect and offer stability to workers or should share risks and promote greater freedom for them.

The person-organization fit (P-O fit) theory provides a theoretical lens that we use as sensitizing concepts to understand this phenomenon in the present study. According to this theory, when an organization and its workers have similar preferences in relation to issues such as values related to employment relations (Cable and Judge, 1996), desirable results tend to be observed, such as job satisfaction (Chen et al., 2016) and performance (Hamstra et al., 2019). Conversely, when these preferences diverge, this misfit tends to produce undesirable results (Felix et al., 2018; De Gieter et al., 2022). According to this theory, therefore, it would be the congruence between the preferences for the values that guide a work relationship, and not the nature of the preferences of the social actors involved, that would determine the results obtained from this relationship (Kulka, 1979). This concept is important for our study, since, in our theory, we explore the preferences that gig-workers and platforms that use algorithmic management have for work relationships that focus more on security or autonomy. More specifically, we focus on understanding the consequences of the occasions when such preferences converge or diverge. To deepen the understanding of these issues, we propose our first research question.

**Research question 1**: What are the consequences of the (in)congruences between the preferences of workers and organizations regarding the dichotomy between worker security and autonomy in the gig-work model?

### 2.5. Paradox theory

Thus far, we have developed the argument that individuals and organizations tend to prioritize security or autonomy in their employment relations (Berger et al., 2019) and that the fit between their preferences would lead to more positive results for both (Cable and Judge, 1996). However, security and autonomy do not necessarily need to be seen as a choice to be made or, in other words, a dilemma. Adopting the lens of paradox theory (Smith, 2014), an individual or an organization can understand that security and autonomy, despite the tension between them, have a relationship of interdependence. In this case, instead of a dilemma, both values would be seen as a paradox, which are contradictory and interrelated dualities that exist simultaneously and persist over time (Smith and Lewis, 2011; Felix, 2020).

Thus, while a dilemma view (Lewis, 2000) proposes a choice between security and autonomy, a paradox view suggests that individuals can achieve security through autonomy (for example, obtaining superior gains to create a financial safety reserve) and autonomy through security (for example, obtaining greater freedom to work daily as a reflection of preserved health due to maximum limits to the work pace). This view was conceptualized by Miron-Spektor et al. (2018) as a paradox mindset, which is the degree to which a social actor accepts dualities and is energized through them. To better understand the effects of this mindset on individuals and organizations in the autonomy-security relationship in the context of gig-work, we propose our second research question.

**Research question 2**: What are the consequences of the existence of a paradox mindset by individuals and organizations regarding the dichotomy between worker security and autonomy in the gig-work model?

## 3. Methods

We conducted a qualitative study based on the procedures of grounded theory (Charmaz, 2014), which is characterized by interactive waves of data collection and analysis, performed with the purpose of developing a theoretical model that explains the phenomenon under analysis from the data. An initial sample of gig-workers was selected, who were interviewed through a research protocol developed from the sensitizing concepts described in the literature review. Subsequently, we followed the procedures of theoretical sampling: we analyzed the collected data, generated memos and first-order codes and asked ourselves what new questions could be asked in the subsequent trips to the field for new data collections and what characteristics of future interviewees would allow us to investigate aspects relevant to the constitution of our theory. Thus, new data were collected and coded, and the first-order codes created were grouped into second-order codes, which were then aggregated so that the initially descriptive codes generated more analytical dimensions (Gioia et al., 2013). This process was stopped after reaching theoretical sufficiency, obtained after a total of six waves of field trips and data analysis. Finally, a theoretical model was constructed, which was presented through propositions regarding how the aggregate dimensions connect to each other and answer the proposed research questions.

### 3.1. Initial and expanded sample

In the first wave of data collection, interviews were conducted with 8 gig-workers from three different organizations, two of which were meal delivery (hereinafter referred to as Alpha and Beta) and one automotive transport (hereinafter referred to as Gamma) company, who showed satisfaction with their relationships with their work organizations. In the second data collection, we used the snowball sampling technique to diversify the profile of respondents in order to include participants who were dissatisfied with the work relationships to which they were subjected. This process led to the completion of 9 new interviews, three with meal delivery workers (Beta), three with general item delivery (Delta) and three with automotive transport (Gamma).

Then, we conducted 10 new interviews, with eight of these individuals working for meal delivery companies (Alpha and Beta) and two for automotive transport companies (Gamma and Epsilon). In this round, one of the interviewees stated that she would start working the following week in an automotive passenger transport company (Omega) that operates through an application platform, offers a fixed minimum wage, its own fleet, life and car insurance, working hours with predefined maximum limits and variable remuneration that allows increasing the value of the fixed monthly remuneration by only 20%. The Omega company prioritizes hiring female and disabled drivers.

Thus, in the fourth field study round, to explore the case of gig-workers who work in an organization that proposes work relationships characterized by higher levels of security, we interviewed 12 workers from that organization (Omega). During these interviews, two of the interviewees reported that they had already worked in an organization with satisfactory levels of autonomy and security. This company (Zeta) offers life insurance for workers who meet certain revenue targets, operates with its own fleet of cars, for which there is insurance, but the remuneration is completely variable and there is no limit on hours worked.

In the fifth data collection round, we interviewed 9 workers from this organization (Omega) who also worked at Zeta to explore cases in which there was both security and autonomy. After this round, we conducted a sixth wave of data collection, in which we interviewed 9 other participants with varied profiles among those who composed the four stages of previous data collection (two from Alpha and two from Beta and one from Delta, Gamma, Epsilon, Omega and Zeta each). As no new codes emerged after this trip to the field, we ended the data collection with a total of 58 respondents, which allowed us a sufficient variation in perceptions, an essential condition for constant comparisons (Charmaz, 2014). Many research participants work for more than one company, but we associated them with the organization in which they more dominantly concentrate their work hours.

In this last round, the nine respondents consensually classified the interviewed organizations as follows (in terms of autonomy and security): Alpha, Beta, Gamma and Epsilon were seen as organizations that offer low security and high autonomy; Omega was interpreted as offering high security and low autonomy; and Zeta was seen as offering intermediate levels of both security and autonomy.

### 3.2. Interviews

The interviews were conducted using a semi-structured script with general questions about the trajectories of the participants. Data collection took place between February 2021 and June 2022. Next, we asked about how they evaluate gig-work, the organizations of this type where they work and their personal preferences specifically regarding issues related to autonomy and security. We then asked them to report specific situations in which they felt satisfied and dissatisfied with the working relationships with these companies, and we asked them to explain why. Last, we asked them to analyse whether this perception has changed over time and whether they understand that the current perception tends to change over time. Based on the fundamentals of grounded theory, the interview script was adjusted between the waves of the iterative data collection and analysis process.

### 3.3. Data analysis

We analyzed the data through the development of memos and first-order codes, that is, expressions in gerund form that describe the reports in a summarized way. According to the principle of constant comparison, we grouped some of these codes into second-order codes, more abstract and of greater theoretical. Next, the second-order codes were analyzed, and some of them were combined to form the aggregate dimensions, which are concepts of greater theoretical scope and which were articulated in the form of theoretical propositions to form the model. In the following, we provide more details about our coding process.

We adopted a two-step coding process for the analysis of our data, as performed by Kreiner et al. (2009). First, we built codes inductively based on the data. Each word, expression, and sentence was considered as a unit of data and, therefore, was subject to coding (Heath and Cowley, 2004). Codes are words or short expressions that are used to synthesize data fragments (Glaser and Strauss, 1967). In this initial process, we documented all first-order codes that we constructed in an emerging codes dictionary.

In the following, two of the three authors read the excerpts from the data that supported each code, then tried to group them independently in the first-order codes that were generated initially. Independent coding processes were also performed in other grounded theories (e.g., Mace and Ward, 2002; Stough and Lee, 2021). The two coders were free to classify all pieces of evidence under one of the codes or ignore them it they understood that there was no correspondence with any of the codes. Next, we performed joint code meetings to analyse the classifications made by both authors and then compared their choices.

Three scenarios could occur as a result of this process: (a) either classify the same evidence under the same first-order code, (b) classify one under one code and eliminate the code for the other, or (c) discard the code for both. In the second case, the author who had not participated in the initial coding process read the excerpt from the interview and acted as a “judge”. This process often generated interesting opportunities for theory building, as disagreements led to analytic dialogues about the meanings of excerpts from the data. This refinement process of the first-order codes was performed at the end of each wave of data collection, what allowed us to improve the emergent codes dictionary. Thus, our coding process counted with multiple perspectives, which helped us to reduce bias in the analysis (Kolb, 2012).

As the first-order codes were more stable, we conducted an analysis with the aim of grouping them into more abstract codes, named second-order codes. The same process of coding verification by two of the authors and subsequent independent analysis by a “judge” was used in the process of generating second-order codes from first-order codes. As the second-order codes were also more stable, we replicated the same process to group them into aggregated dimensions, which are even more abstract codes, with greater theoretical reach, and that were generated from the second-order codes. This process of transforming first-order codes into second-order codes and, later, into aggregated dimensions, is also found in other studies that adopted a grounded theory approach (Byron and Laurence, 2015; Felix and Cavazotte, 2019).

**Figure 1:**
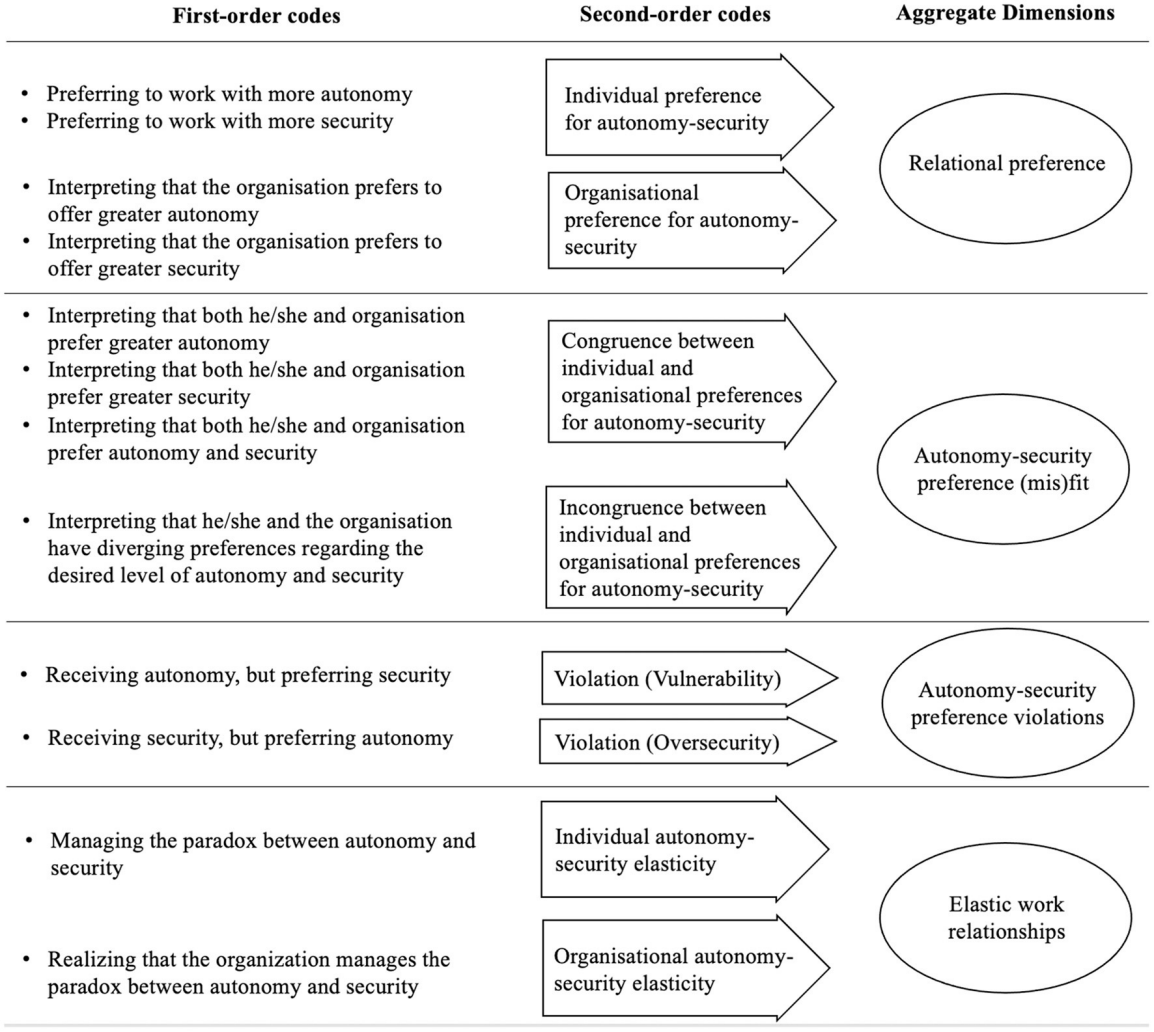
General structure of derivation of codes and dimensions.

The process of data collection and analysis was completed when we reached theoretical saturation (Glaser and Strauss, 1967), i.e., when new waves of data collection and analysis didn't lead to new codes or dimensions. We identified theoretical saturation when we realized, after the fifty-first interview, that we were not generating new codes anymore. Figure 1 summarizes the structure of the codes and aggregate dimensions that were generated after the coding of the interviews.

## 4. Results

**Figure 2:**
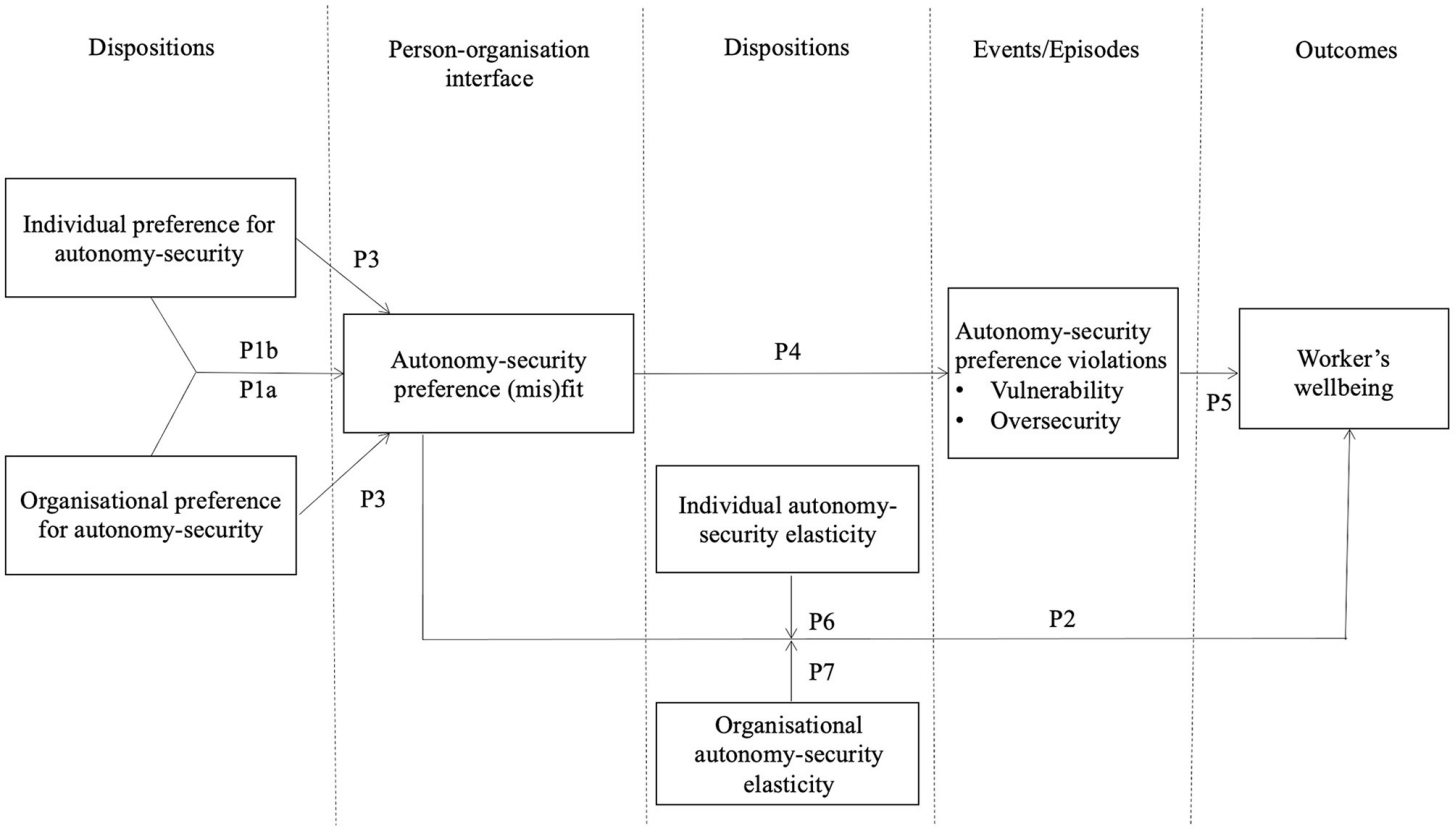
A grounded theory of gig-worker wellbeing.

In this section, we present the results, systematized by means of a theoretical model, in which derived categories are related through theoretical propositions. Figure 2 illustrates the model to be presented.

### 4.1. Understanding the congruences between preferences and their consequences

Our data allowed us to identify that the interviewed workers varied in terms of their relational preferences, i.e., their preference for working in interactions characterized by more autonomy or by more security. We used the code “*Preferring to work with more autonomy”* and “*Preferring to work with more security”* to categorize the statements of the interviewees that fit the two situations. Below we present examples that substantiated both categories, respectively.

“This thing with a formal contract is an illusion. I prefer a thousand times more to be more autonomous, to be able to choose the time when I go to work. I like to choose whether I will work more and earn more in a month, when I need it, even if I have less stability” (male, 32 years old, Beta).

“Look, I would prefer that the company hire me, that I was an employee, with a formal contract, or that I had more guarantees. I prefer to have more stability, because this way we can plan” (female, 28 years old, Epsilon).

While in the first case the worker reported preferring to work with more freedom to choose his work schedules and monthly income flow, in the second case, there was a preference for more stable and secure relationships with more guarantees. Similarly, respondents also reported that, in their interpretation, the companies for which they work vary in terms of the degree to which they propose relationships more characterized by autonomy or security. The codes “*Interpreting that the company prefers to offer greater autonomy”* and “*Interpreting that the company prefers to offer greater security”* were used to categorize the data that supported this understanding. Next, we present empirical evidence for both codes.

“Here, without a doubt, they prefer to give you more freedom, make less commitments to give you stability, so much so that they do not accept a formal contract, but they pay when you work a lot and they don't get upset with those who want to spend a few days without accessing the application” (male, 52 years old, Gamma).

“This company is different from most application companies because here they pay you to be stopped in the street, waiting for a call. But if there's a day with a lot of calls, you will earn a fixed amount, you don't earn much more for it. They prefer more security” (female, 35 years old, Omega).

The interpretation that the company tends to prefer more relationships with more autonomy was dominant in our data. However, even among these, the perception of the point of the continuum between autonomy and security in which the preference of a company is located varied. For example, according to some participants, while company Alpha requires workers to work on weekends, Gamma does not. However, both are seen as organizations that prefer relationships based on greater autonomy. All cases in which the respondents reported that they understand that the company prefers greater security refer to the company Omega.

In some cases, respondents reported that their personal dispositions for greater autonomy or security and the dispositions they perceived by the companies responsible for the gig-work platforms were compatible. Thus, in some cases, workers indicated perceiving a preference by both for greater autonomy, and in others, the perception was that both preferred greater security. For both cases, we used the code “*Autonomy-Security Preference Fit”* to represent this congruence of preferences. The following reports represent, respectively, a perception of fit of preferences for autonomy and fit of preferences for security.

“The fact that the company has not registered me, that we have this power to make money at our pace, suits me. I never did well working in the office, during business hours, punching a card. So, what I want is what the company wants, it combines well (...). And this makes me feel good at work, I feel I have more wellbeing when this happens” (man, 28 years old, Alpha) (*Autonomy Preference Fit)*.

“The good thing is that me and Omega both prefer more security. I came here to work precisely because of that, I had never worked in an application company before (...). For me to feel good, it has to be like this, with more benefits; if there's too much insecurity, it's impossible” (woman, 47 years old, Omega) (*Security Preference Fit)*.

Our first research question refers to the consequences of the (in)congruences between the preferences of workers and organizations regarding the dichotomy between worker security and autonomy in the gig-work model. Both reports suggested that when individuals and organizations had congruent preferences in terms of the degree to which work relationships are characterized by more autonomy or by more security, such fit tended to lead to a greater generalized state of gig-worker wellbeing. Thus, we suggest that by directing our gaze to the interface of the preferences of both, we obtain a broader and interactional view regarding the phenomenon of the construction of gig-worker wellbeing. This evidence leads us to the first propositions of this study.

**Proposition 1a:** When individuals and organizations have a preference for security, there is Security Preference Fit.

**Proposition 1b:** When individuals and organizations have a preference for autonomy, there is Autonomy Preference Fit.

**Proposition 2:** Autonomy/Security Preference Fit has a positive effect on worker wellbeing.

### 4.2. Understanding the incongruences between preferences and their consequences

Several of the study participants reported that they would prefer that work relationships have a different level of autonomy or security than that proposed by the company where they work *Autonomy-Security Preferences Misfit*). In most cases, the respondents reported that they would like higher levels of security, while the company responsible for the platform proposes relationships more guided by autonomy. However, in others, especially among Omega workers, there were reports that there was a desire for greater autonomy, while the company offered a relationship more grounded in security. This misfit of preferences led to episodes of dissatisfaction in which the workers reported that their preferences regarding autonomy or security were not present in the relationship with the company. We coded these cases as “*Autonomy/Security Preference Violations*”, which can occur by “Vulnerability” (when the worker desired greater security than that found) or “Oversecurity” (when the worker desired greater autonomy than that proposed by the company). Both were coded using participants' own words (*in-vivo* codes). Next, we present reports that illustrate Autonomy-Security Preference Misfits, their respective and consequent violations by vulnerability and oversecurity, and their impacts on worker wellbeing.

“What kills here is this more uncertain thing, of not knowing if there will be a call. If you get sick, nothing comes into the bank. I once crashed the car and spent five month's profit to fix it. It is complicated, I wish there was more security for us to work, but there is not” (male, 23 years old, Gamma) (*Security Preference Violation - Vulnerability)*.

“I wanted (...) to earn more according to the number of drives, to work with a more flexible schedule. Here, it is not like that, you are sometimes stopped at times when nothing comes up. One day I had a lot of energy to continue getting drives and I needed money, but I had to stop because the shift ended, another driver came to drive the car (...). It happens all the time. I keep feeling trapped” (female, 24 years old, Omega). (*Autonomy Preference Violation - Oversecurity)*.

The reports of both respondents described events in which they felt uncomfortable with the level of autonomy or security found in the relationship with the companies. According to the statements of the study participants, these episodic violations of expectations, when they occur repeatedly and in the long term, reduce their sense of wellbeing. Thus, we propose the following:

**Proposition 3:** When individual and organization have different preferences in terms of security/autonomy, there is Autonomy/Security Preference Misfit.

**Proposition 4:** Autonomy/Security Preference Misfit leads to Autonomy/Security Preference Violations, which may occur through a) Vulnerability or b) Oversecurity.

**Proposition 5:** When repeated in the long term, Autonomy/Security Preference Violations have a negative effect on worker wellbeing.

### 4.3. Beyond “or”: The role of the paradox mindset

Although most of the study participants reported that they or the organizations with which they worked have a preference that tends more toward worker autonomy or security, this does not mean that they interpret autonomy and security as a dilemma (either/or). For some interviewees, although there is a preference for working relationships more guided by autonomy or security, both needs are seen as essential for the sustainable development of the career and of their wellbeing. When examining some interviews with this paradox mindset regarding the dichotomy between autonomy and security, we created and sought to answer our second research question. In this section, therefore, we discuss how the wellbeing of gig-workers can be influenced by the existence of a paradox mindset by individuals and organizations.

We call “Individual autonomy-security elasticity” the mindset that some individuals present, according to which autonomy and security in work relationships are values considered equally important and, therefore, should be pursued simultaneously. In the following example, a gig-worker reported that he and the organization for which he works have a relationship more characterized by promotion of freedom and autonomy. Thus, because he has a high elasticity between autonomy and security, the gig-worker uses the higher earnings obtained as a result of a relationship in which his autonomy and productivity are remunerated to pay for his security. Thus, such elasticity allowed him to minimize any negative consequences of relationships characterized by autonomy through proactive planning to attain security. The following excerpt from the interview with a worker from Delta Company shows this understanding.

“Look, I enjoy working more freely, but I need to feel supported, so I try to use freedom to pay for my protection. I started a savings fund for when I have to fix the motorcycle, for health problems and everything. I'm able to do this because I earn more than I earned in a secure job. So, if you take a longer period, one or two years, and analyse how much money goes in and out, I earn more today. So I know that the company will not protect me, but I prefer it that way” (male, 40 years old, Delta).

In general, although individuals who reported experiencing autonomy preference misfit reported satisfactory wellbeing, in some cases, drops in long-term wellbeing were reported due to the existence of excessive workload and carelessness with health. We note that for individuals with higher levels of individual autonomy-security elasticity, the positive benefits of autonomy preference fit were expanded, given that the paradoxical mindset led them to proactively establish limits to their work pace, for example. Thus, they were less subject to the risk of abusing the freedom to determine their workload and began to reap positive results from both autonomy and security. The following case illustrates this finding.

“For me, what matters is that I can work as much as I want, so I spent a year working more or less 18 hours a day, every day of the week. But one day I crashed, I slept at the wheel. The company gave me all the autonomy, it was what I wanted, but I screwed myself up. It was not possible to sustain that rhythm (...). Today, I know I need both freedom and security” (male, 21 years old, Zeta).

In some cases of Omega workers who experience security preference fit in the relationship with this company, the individual autonomy-security elasticity also influenced the way such fit relates to worker wellbeing. In general, as previously explained, when both the individual and the organization showed a preference for relationships more characterized by security, this fit tended to lead to the perception of greater wellbeing. However, some participants reported that this configuration settled them (made them comfortable with the situation), which led to a reduction in the increase in their wellbeing. According to them, this reduction occurred because some felt that the relationship based on security was accompanied by difficulty in achieving objective career success.

However, some individuals claimed to have the ability to deal simultaneously with their needs for security and autonomy. In these cases, the security obtained in the relationship with Omega was seen as a means for them to try to engage in riskier activities, and more financially rewarding, in other domains. Thus, when coping well with the conciliation of the needs for security and autonomy, some workers, among those who experienced security preference fit, did not give in to recurrent settling. Therefore, for those who showed elasticity between autonomy and security, there was a more intense perception of wellbeing. The following case illustrates this understanding.

“Me and the company are more likely to prefer security, and I know that this makes us more settled. I see colleagues live the life of leisure, but this does not work for me, I do not settle. I have a certain tranquility here that I did not have in Gamma, and I use this tranquility to fund my adventures as an entrepreneur. Here, I make enough money, I know it will not be lacking. Then, I use my free time and some of the money I save to invest in some attempts, and it is in these that I want to be freer and try to make more money” (female, 28 years old, Omega).

Thus, we propose that:

**Proposition 6:** Individual Autonomy-Security Elasticity intensifies the positive relationship between Autonomy-Security Preference Fit and worker wellbeing.

The mindset of paradox between autonomy and security was noted not only among workers but also in the perceived practices of the service provider platforms. While most companies were seen as adopting practices that favored either autonomy (Alpha, Beta, Gamma and Epsilon) or security (Omega), company Zeta was perceived as adopting paradoxical practices that aimed to promote both worker autonomy and security. Thus, we codify this form of paradox mindset as “Organizational Autonomy-Security Elasticity”.

Similar to what we identified in the individual level, Organizational Autonomy-Security Elasticity also enhanced the positive effects of Autonomy-Security Preference Fit on worker wellbeing. Because company Zeta proposes a work relationship in which it offers its own fleet of cars and car and life insurance (security) but with completely variable remuneration and without limiting work hours (autonomy), it can offer satisfactory support to individual needs for both security and autonomy.

As stated above, individuals who prefer relationships based on autonomy stated perceiving greater risks of working without trying to promote their own security (without paying for life or car insurance, for example). Thus, when working in an organization that seeks to establish criteria for workers to be entitled to more security factors through higher productivity, the risk of having their wellbeing decreased were reported as lower. The following interview excerpt illustrates this understanding.

“There are companies that give you zero security and force you to work on weekends and at least two nights or evenings during the week for you to stay in the system. There is another where everything is more secure, but you don't earn enough. Here, it is the most balanced I have ever seen, there is autonomy and security. I, for example, tend to want freedom, but I also tend to not stop working when my body needs it, and I don't save. Here, I end up getting stronger on this side too, of getting more security, the company forces me to stop after a while on the street and gives me freedom only as long as it knows I will not harm myself” (male, 43 years old), Zeta).

Similarly, workers who prefer relationships based on security, when working in an organization with greater elasticity between security and autonomy, reported that they perceive less chance of experiencing the negative side of security: settling. According to the interviewees, this is because the organization creates incentives for workers to exercise their freedom and achieve goals that will ensure the desired security. Thus, they avoid settling behaviors that would distance them from objective career outcomes that were reported as important for their perception of wellbeing. In the following report, there is evidence for this understanding.

“Here, there are these two things, autonomy and security. I like the bond, feeling protected, knowing that I won't be wanting. This is good, the company offers all this, predictability, but at the same time they set goals that make me want to produce more, get five stars from the customer. I become more ambitious, but in a way that is positive, is sustainable. I still have free time to live well, but today I have the financial means to afford leisure for my family (...) And wellbeing for me involves this” (male, 37 years old, Zeta).

Thus, the following proposition is presented:

**Proposition 7:** Organizational Autonomy-Security Elasticity intensifies the positive relationship between Autonomy-Security Preference Fit and Worker Wellbeing.

## 5. Discussion and conclusions

Although previous studies have discussed the benefits (Hall and Krueger, 2018; Berger et al., 2019) and harms (Malin and Chandler, 2017; Koutsimpogiorgos et al., 2020) of algorithmic management for gig-workers, a discussion is needed that considers these effects simultaneously and that considers the point of view of gig-workers. In this study, by adopting a perspective that considered the fit between the preferences of workers and gig-work platforms, we added to the literature a look at the interface between the interests of both social actors (Kulka, 1979). As a result, we found not only harms or benefits to workers but also a complex dynamic that takes into account the positive (and negative) effects of a (mis)fit of interests between worker and organization. Unlike studies that adopt a predominantly critical stance on the effects of gig-work on worker wellbeing (Malin and Chandler, 2017; e.g., Anwar and Graham, 2019), we identified reports that suggest that several workers who desire a similar degree of autonomy/security to what is offered by the organization tend to report the perception of wellbeing at work.

However, not everything was found to be a matter of fit. This positive perception of wellbeing in the interviewees' report was accompanied by an interpretation that such fit may represent a trap for the wellbeing of gig-workers, especially when the worker has a tendency to see autonomy and security as a choice/dilemma and not a paradox. This view is echoed in the concept of paradox mindset (Miron-Spektor et al., 2018), which suggests that, by understanding the interdependence relationship between two conflicting elements, individuals tend to better manage the tension involved between the polarities of a paradox. Thus, we introduced, at the individual and organizational levels of analysis, the concept of elasticity between autonomy and security, which we believe to be promising as an additional alternative, together with the study by Berger et al. (2019), for the adoption of a non-polarized discussion between the benefits and harms of algorithmic management for the wellbeing of gig-workers.

### 5.1. Theoretical implications

This study has implications for the literature on the impacts of algorithmic management on worker wellbeing. Our study draws attention to an integrated discussion of the benefits and harms of algorithmic management, which allows for overcoming a polarized view in which it would be seen only as beneficial or harmful to workers (Duggan et al., 2020). In addition, by adopting a fit perspective for the gig-worker wellbeing phenomenon (Cable and Judge, 1996), we also draw attention to the fact that the construction of a sense of wellbeing may result not only from the content of the relationship itself but also from a congruence of interests between those involved. Additionally, by highlighting the possibilities of workers working excessively or settling in cases of autonomy-security preference fit, respectively, our theory shows the limitations of the fit perspective for the phenomenon studied here.

Our study also contributes to the literature on paradoxes in the work context. So far, studies on paradoxes have focused mainly on two dimensions: the organizational and the individual. In the organizational dimension, the characteristics of organizations that simultaneously manage conflicting goals such as organizing, performing, learning, and belonging are explored (Smith and Lewis, 2011; Felix, 2020). In the individual dimension, the paradox mentality has been explored, which is a lens for the interpretation of reality through which individuals value, accept, and feel comfortable with tensions (Miron-Spektor et al., 2018; Cavalcanti et al., 2022). Our model allows advancing this discussion by exploring not merely organizational goals, but rather how individual paradoxical goals can be achieved through a paradoxical relationship between individuals and organizations. We also advance this discussion by exploring this phenomenon in a particular research context/industry (gig economy/gig workers).

### 5.2. Limitations and future studies

The present study has some limitations, and future studies may be conducted to overcome them. First, the data were collected in interviews that occurred at a specific time and therefore do not provide a longitudinal view of the dynamics between individuals and organizations between security and autonomy. As the theory about paradoxes suggests that the perspective of time is relevant for understanding how their polarities are managed over time (sequentially or simultaneously) (Lewis, 2000), longitudinal studies could help fill this gap.

Second, the study did not explore the factors that led to the emergence of gig-work platforms that propose a model with higher levels of worker security (Zeta and Omega). Future case studies could explore the role of individual, group or societal articulations, of an economic or ideological nature, and in face-to-face or digital interactions (Maffie, 2020) in the emergence of these organizations. Just as the company Omega was created with the purpose of offering fairer working conditions for women and other groups at a historical disadvantage, it is possible that similar phenomena are observed in other cultures.

### 5.3. Practical implications

The study shows gig-workers ways to find greater wellbeing at work. For this, they should seek to identify their work-related values (more oriented toward autonomy and/or security) and seek to develop relationships with organizations with which they have an alignment of preferences, when these exist in the market. In addition, public manifestations of dissatisfaction with working conditions can help consumers to become more sensitive to their demands.

As gig-work platforms that offer greater security to workers are not common, it is also suggested that new organizational forms, such as worker cooperatives or non-profit organizations, with proposals for resistance to abuses commonly found in the gig economy's work relationships, be implemented through social entrepreneurship strategies. For example, gig-workers could get together and create cooperatives to develop an application that manages its performance through algorithmic management. Also, they could apply part of the financial gains toward offering benefits and greater security for the cooperative members involved.

For existing platforms using algorithmic management, we suggest offering contract options in which workers can enjoy greater benefits that bring them greater security. One way to make it possible to offer such benefits would be to offer the consumer the option of selecting a service contracting option that would allow the gig-worker to better reconcile autonomy and security. It is reasonable to imagine that, given the growth of the phenomenon of conscious consumption (Kingston, 2021), many consumers would agree to pay higher amounts that would allow for a fairer work relationship.

## Data availability statement

The raw data supporting the conclusions of this article will be made available by the authors, without undue reservation.

## Ethics statement

The studies involving human participants were reviewed and approved by the Programa de Pós Graduação em Administração/Fucape Business School. This study was carried out in accordance with the recommendations of the FBSR Guidelines, Ethics Committee at Fucape Business School with written informed consent from all subjects. All subjects gave written informed consent in accordance with the Declaration of Helsinki. The protocol was approved by the Ethics Committee at Fucape Business School. The patients/participants provided their written informed consent to participate in this study.

## Author contributions

BF and DD has collected all the data. BF, DD, and VN analyzed the data and wrote the manuscript. All authors contributed to the article and approved the submitted version.
